# Implications of Hydrogen Sulfide in Development of Pulmonary Hypertension

**DOI:** 10.3390/biom12060772

**Published:** 2022-06-01

**Authors:** Yan Sun, Chaoshu Tang, Hongfang Jin, Junbao Du

**Affiliations:** 1 Department of Pediatrics, Peking University First Hospital, Beijing 100034, China; sunyan2007bjmu@163.com; 2 Key Laboratory of Molecular Cardiovascular Sciences, Ministry of Education, Beijing 100191, China; tangchaoshu@263.net.cn; 3 Health Science Centre, Department of Physiology and Pathophysiology, Peking University, Beijing 100191, China

**Keywords:** hydrogen sulfide, pulmonary hypertension, remodeling, pulmonary artery

## Abstract

The pathological mechanisms underlying pulmonary hypertension (PH), as well as its treatment strategy, are crucial issues in this field. This review aimed to summarize the pathological mechanisms by which the hydrogen sulfide (H_2_S) pathway contributes to PH development and its future implications. The data in this review were obtained from Medline and PubMed sources up to 2022 using the search terms "hydrogen sulfide" and "pulmonary hypertension". In the review, we discussed the significance of endogenous H_2_S pathway alteration in PH development and showed the advance of the role of H_2_S as the third gasotransmitter in the mechanisms for hypoxic PH, monocrotaline-induced PH, high blood flow-induced PH, and congenital heart disease-associated PH. Notably, H_2_S plays a crucial role in the development of PH via certain mechanisms, such as inhibiting the proliferation of pulmonary artery smooth muscle cells, suppressing the inflammation and oxidative stress of pulmonary artery endothelial cells, inducing pulmonary artery smooth muscle cell apoptosis, and interacting with other gaseous signaling pathways. Recently, a variety of H_2_S donors were developed, including naturally occurring donors and synthetic H_2_S donors. Therefore, understanding the role of H_2_S in PH development may help in further exploring novel potential therapeutic targets of PH.

## 1. Introduction

Pulmonary hypertension (PH) is regarded as a fatal pathophysiological process, with abnormally elevated pulmonary artery pressure (PAP) and even right ventricular dysfunction failure in some cases [1]. A mean PAP of up to 20 mmHg at sea level and a determination of right heart catheterization at rest are the criteria used to diagnose PH [2]. PH affects approximately 1% of the global population, i.e., a prevalence of approximately 25 cases per 1 million people [2,3,4,5]. Additionally, it usually has a poor prognosis, with high disability and mortality rates. Once diagnosed, it is sometimes difficult to cure and can be life-threatening in severe cases [6,7]. Therefore, it is extremely important to explore the mechanisms of PH development. 

The endogenous gasotransmitters, including nitric oxide (NO), carbon monoxide (CO), hydrogen sulfide (H_2_S), and sulfur dioxide (SO_2_) [8,9,10,11,12,13,14,15,16,17,18], with unique properties, such as rapid generation, fast transmission, extensive functions, and short half-lives, play vital roles in the pathogenesis of PH [19,20,21]. Great progress has been made in understanding the involvement of these gasotransmitters in the pathogenesis of PH [17,18,21]. 

**Figure 1:**
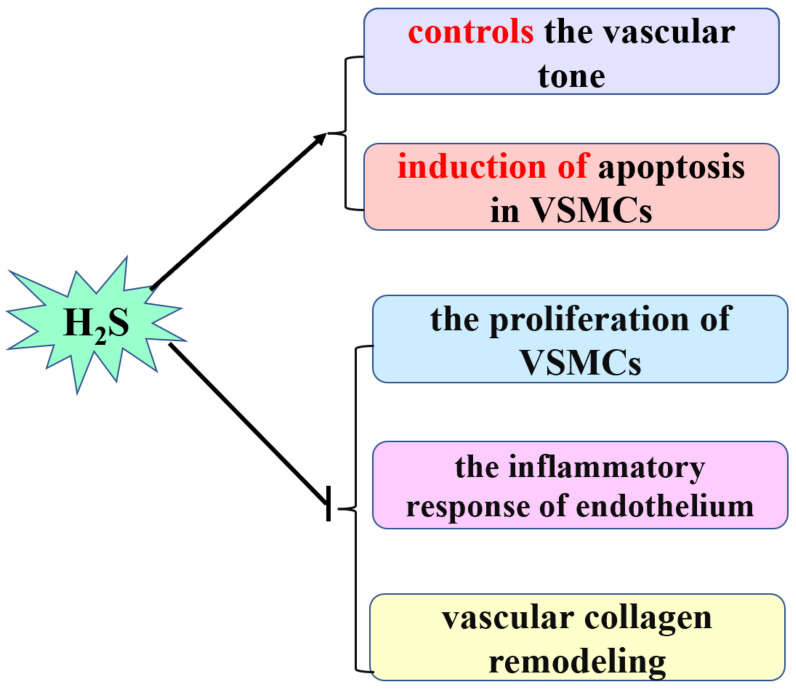
The cardiovascular physiological effect of H_2_S. H_2_S: hydrogen sulfide; VSMCs: vascular smooth muscle cells.

Endogenous H_2_S, a third gasotransmitter, is involved in the development of a variety of cardiovascular diseases [17,18,22,23,24]. H_2_S is mainly catalyzed by enzymatic pathways, and it is also regulated by several metabolic pathways. It exerts important cardiovascular physiological effects [8,10,13]. For instance, it controls the vascular tone, reduces blood pressure and PAP, inhibits the vascular smooth muscle cell (VSMC) proliferation, regulates the endothelial inflammatory response, induces VSMC apoptosis, and inhibits vascular collagen remodeling [17,18] (Figure 1). 

The downregulated endogenous H_2_S pathway has been observed in cardiovascular and pulmonary vascular diseases, such as PH, hypertension, atherosclerosis, ischemic myocardium, cardiac injury, heart failure, and septic shock [10,13,17,18,21]. However, exogenous supplementation with H_2_S or H_2_S donors can halt the progression of these cardiovascular diseases. 

In the present review article, we discussed the biological origin of the endogenous H_2_S in cardiovascular cells, and the role of endogenous H_2_S in the development of PH, as well as the mechanisms. In addition, we discussed the crucial role of H_2_S in the different types of PH, including hypoxic PH (HPH), monocrotaline (MCT)-induced PH, high blood flow-induced PH, congenital heart disease (CHD)-associated PH, and chronic obstructive pulmonary disease (COPD)-associated PH.

## 2. Biological Origin of H_2_S in the Cell

H_2_S is regarded as an important gasotransmitter in the regulation of various biological and pathophysiological processes [17,18,25]. H_2_S shows an increased solubility in lipids and aqueous solution, with an efficient capability of crossing plasma membranes. The generation pathways of endogenous H_2_S in the cell include the enzymatic pathway and non-enzymatic pathway. It is preliminarily produced by the enzyme-catalyzed reaction in the cytoplasm, using L-cysteine (L-Cys) as a substrate. The key enzymes mainly consist of cystathionine-γ-lyase (CSE), cystathionine-β-synthase (CBS), 3-mercaptopyruvate sulfur transferase (3-MST), and cysteine aminotransferase (CAT) [17,25]. In the cytoplasm, H_2_S is catalyzed by CSE and CBS with substrates L-cysteine and L-homocysteine (Hcy). 3-MST, in combination with CAT to generate H_2_S from L-cysteine, is demonstrated in both cytoplasm and mitochondria. It is also reported that 3-MST can generate H_2_S with substrate D-cysteine, in the coordination of D-amino acid oxidase. The expressions of these key enzymes are tissue-specific. CSE is abundant in the thoracic aorta, liver, portal vein, ileum, and non-vasculature. CBS is mainly expressed in the brain, kidney, and liver. 3-MST plays a role in regulating H_2_S in the aorta, kidney, brain, and liver. CSE exerts a key effect on H_2_S generation in the cardiovascular system. However, it is different from the related enzymatic generation pathways, and the non-enzymatic reaction of H_2_S production is partially catalyzed by the synergistic action of VitB6 and iron with cysteine as a substrate, in the heart, lung, spleen, muscles, plasma, and bone marrow, as well as especially in erythrocytes [17,25].

## 3. Role of H_2_S in HPH

The pathophysiological processes of HPH mainly include progressive pulmonary vasoconstriction, pulmonary vascular inflammation, pulmonary vascular oxidative stress, and pulmonary vascular structural remodeling [1,5,6]. In early 2003 [26], our team, for the first time, showed the significance of H_2_S in pulmonary circulation and reported that H_2_S levels in lung tissues and plasma were reduced. Moreover, the expression and activity of CSE were inhibited in the pulmonary artery tissues and lung tissues of HPH rats. Interestingly, after the supplementation with an H_2_S donor, PAP was significantly reduced, and the pulmonary vascular structural remodeling was alleviated [26]. Studies also elucidated that endogenous H_2_S inhibits the formation of HPH, and the downregulation of the endogenous H_2_S pathway is a key mechanism for the progression of HPH [27,28,29,30]. Therefore, the insufficient H_2_S production promotes HPH.

**Figure 2:**
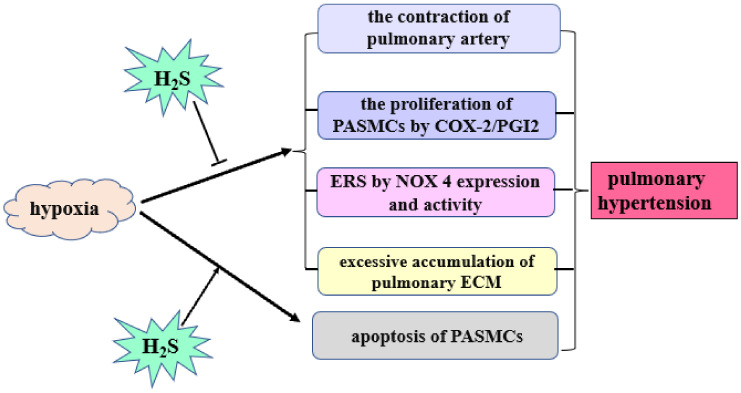
The role of H_2_S in hypoxic pulmonary artery hypertension. H_2_S: hydrogen sulfide; PASMCs: pulmonary arterial smooth muscle cells; COX-2: cyclooxygenase-2; PGI_2_: prostaglandin; ERS: endoplasmic reticulum stress; NOX4: nicotinamide adenine dinucleotide phosphate oxidase 4; ECM: extracellular matrix.

H_2_S controls HPH by employing the following mechanisms [26,27,28,29,30,31,32,33] (Figure 2): (1) relaxing vascular smooth muscles by mainly opening the K_ATP_ channel on VSMCs; (2) directly repressing the hypoxic pulmonary artery SMC (PASMC) proliferation and inhibiting hypoxia-induced cell proliferation through the upregulation of cyclooxygenase-2/prostaglandin; (3) promoting hypoxia-induced apoptosis of PASMCs; (4) effectively inhibiting endoplasmic reticulum stress via suppressing the reduced nicotinamide adenine dinucleotide phosphate oxidase 4 (NOX-4) expression and activity [30]; and (5) inhibiting the pulmonary extracellular matrix (ECM) accumulation. Of note, collagen and elastin gradually accumulate in an atypical manner in the adventitia of pulmonary arterioles in HPH [28]. In a hypoxic rat model, exogenous H_2_S donors can reduce the production of pulmonary artery collagen, elastin, and transforming growth factor-β3 and inhibit the expression of procollagen mRNA. These results suggest that endogenous H_2_S can inhibit collagen and elastin synthesis, promote collagen degradation, and alleviate hypoxic pulmonary vascular remodeling [28]. 

Clinically, HPH is an important pathological change in patients with COPD partially due to airway obstruction-induced hypoxia. Yuan et al. found that serum H_2_S concentration in patients suffering from the acute exacerbation of COPD (AECOPD) with PH was lower than that in patients suffering from AECOPD without PH and healthy population [34]. Furthermore, the serum H_2_S in the AECOPD patients was negatively correlated with pulmonary artery systolic pressure (PASP). Similarly, the expression of CSE in the pulmonary artery of patients suffering from stable COPD with PH was lower than that in patients suffering from stable COPD without PH and healthy controls. Mechanistically, the upregulated NOX4/reactive oxygen species (NOX4/ROS) pathway might be involved in the possible mechanisms by which the deficiency of endogenous H_2_S contributes to the development of COPD-associated PH [34].

## 4. Role of H_2_S in MCT-Induced PH

PH is a progressive disease due to increased PAP and right ventricular failure [35]. MCT-induced PH is used as a classical animal model of PH in the experiment [36,37,38]. MCT, a toxic alkaloid, can lead to the proliferation of PASMC and inflammation of endothelial cells, even causing right heart dysfunction resulting from right cardiac overloading [36]. Feng et al. [37] showed that the endogenous H_2_S/CSE was downregulated in rats suffering from MCT-induced PH, and the supplement of H_2_S donor reduced PAP and relieved vascular structural remodeling, thus significantly improving the progression of PH. Moreover, this study demonstrated that the downregulated endogenous H_2_S/CSE pathway was related to pulmonary vascular inflammation in pulmonary hypertensive rats. Therefore, H_2_S exhibited a protective effect on MCT-induced PH.

Other studies revealed the underlying mechanisms by which H_2_S was involved in the MCT-induced PH [37,38,39,40,41]. H_2_S inhibited the inflammation of pulmonary arterial endothelial cells and prevented pulmonary vascular remodeling in MCT-induced PH, probably through inhibiting the nuclear factor kappa B (NF-κB) signaling pathway and endothelial–mesenchymal transition in pulmonary arteries [37]. Furthermore, in vivo and in vitro findings demonstrated that endogenous H_2_S directly deactivated the inhibitor of the κB kinase subunit β (IKKβ) by sulfhydrating its Cys179 to prevent the activation of the NF-κB pathway and subsequently control the inflammation of pulmonary artery endothelial cells in PH [39]. In addition, H_2_S controlled MCT-induced PH in rats by inhibiting the aggregation and degranulation of mast cells and the release of interleukin-6 [41]. Endothelial-to-mesenchymal transition (EndMT) plays an important role in PH. The investigators indicated a beneficial effect of H_2_S on PH development via inhibiting the NF-κB pathway and the subsequent pulmonary artery EndMT [40].

## 5. Role of H_2_S in High Pulmonary Blood Flow-Induced PH

High pulmonary blood flow-induced PH is a common complication of CHD in patients with a left-to-right shunt [42]. The severity of PH progression closely affects the timing of surgeries, their success rate, and post-operative prognosis. In experimental studies on rats, an animal model of high pulmonary blood flow-induced PH was successfully developed by performing an experimental operation to create an abdominal aorta/inferior vena cava shunt. Li et al. [43,44] reported that the H_2_S/CSE pathway of the lung tissue in rats was increased following 4 weeks of shunting. However, the H_2_S/CSE of the lung tissues of shunt rats after 11 weeks was downregulated. At the same time, PASP was markedly raised, and pulmonary vascular structural remodeling developed in the shunt rats. After exogenous H_2_S donor supplementation in shunt rats, the pulmonary vascular remodeling was reduced, and the PASP was successfully decreased.

We showed that H_2_S played its regulatory role in PH induced by increased pulmonary blood flow via several mechanisms [42,43,44,45,46,47]. In one study, H_2_S inhibited the proliferation of PASMCs through mitogen-activated protein kinase/extracellular signal-regulated kinase signaling to alleviate the pulmonary vascular structural remodeling and PH induced by high pulmonary blood flow in rat models [44]. In addition, it inhibited the pulmonary artery inflammatory response of rats with increased pulmonary blood flow, via downregulation of the NF-κB pathway. Other studies also showed that H_2_S promoted collagen degradation in the pulmonary artery walls and reduced the accumulation of ECM in the pulmonary vascular structural remodeling and PH caused by increased pulmonary blood flow [17,45]. Interestingly, these studies showed that H_2_S regulated the production of vasoactive peptides, such as endothelin-1 (ET-1), atrial natriuretic peptide (ANP), calcitonin gene-related peptide (CGRP), and pro-adrenomedullin peptide (PAMP) to regulate the pulmonary hemodynamics and structure [48]. Furthermore, it inhibited the production of endogenous vasoconstrictors, such as ET-1, ANP, and CGRP but promoted the plasma vasoactive PAMP levels to relax blood vessels and relieve PH [48].

Of note, the interaction between H_2_S/CSE and NO/nitric oxide synthase (NOS) pathways was involved in the development of high pulmonary blood flow-induced pulmonary vascular structural remodeling and PH. Wang et al. found that after 11 weeks of abdominal aorta-inferior cava vein shunting operation, high pulmonary blood flow-induced pulmonary vascular structural remodeling and PH developed in association with a down-regulated H_2_S/CSE pathway. While, for shunt rats administrated with L-arginine, a substrate of NOS, the H_2_S/CSE pathway was markedly upregulated in the shunt rats with L-arginine treatment, and at the same time, the pulmonary artery pressure was significantly decreased in comparison to those in the shunt rats without L-arginine treatment. The above results suggested that the upregulated endogenous H_2_S might partly contribute to the inhibitory effect of L-arginine on the high blood flow-induced PH [42].

## 6. Role of H_2_S in PH Associated with CHD

PH is a common complication of CHD [49]. Sun et al. [49] reported that decreased H_2_S and increased Hcy concentrations were correlated with PH in patients with CHD. The study indicated that the plasma Hcy contents and the H_2_S concentration yielded good sensitivity and specificity to predict obstructive PH in CHD cases, respectively, indicating that Hcy and H_2_S are potential diagnostic biomarkers. Tan et al. [50] also reported that H_2_S levels have an important predictive value for the prognosis of CHD. They showed that the endogenous H_2_S concentration was negatively correlated with the mechanical ventilation duration, duration of stay in ICU, and maximum vasoactive drug scoring value at 24 and 48 h following cardiac surgery, respectively. The results suggested that the endogenous H_2_S levels had a potential clinical significance in the prediction of the prognosis of CHD cases after cardiac surgery.

## 7. Conclusions and Perspectives

Endogenous H_2_S as a third gaseous molecule plays a crucial part in the pathophysiology responsible for PH. H_2_S attenuates the vascular endothelial cell inflammatory response, inhibits PASMC proliferation, modulates vascular cell apoptosis and inhibits collagen remodeling, opens the K_ATP_ channel to relax pulmonary vessels [11,17,18], and interacts with CO and NO signaling pathways to exert vascular function and maintain normal pulmonary circulation [19]. Under certain pathologic stimuli, the endogenous H_2_S pathway is downregulated, thus inducing the development of PH. 

Further understanding the involvement of the H_2_S pathway and the molecular mechanisms underlying the development of PH, as well as its vascular function regarding the pulmonary vessels, would attract great interest for the exploration of novel potential therapeutic targets of PH in future studies. The studies show that H_2_S plays a protective part in the development of PH and might be a target for a new treatment strategy with H_2_S-releasing molecules [51]. The potential therapeutic effect is mainly established in H_2_S supplementation experiments using H_2_S donors. The most widely used H_2_S donors are NaHS and Na_2_S [19,51,52,53,54,55]. They have several advantages, such as being inexpensive, water-soluble, and having the ability to rapidly release a large amount of H_2_S under physiological conditions. While GYY4137 or dithiolthione compounds work as slow-releasing H_2_S donors, are actively developed, and exhibit promising effects on cardiovascular diseases [19,56,57], some other H_2_S donors have been demonstrated to protect against cardiac dysfunction, vascular remodeling, and PH. Recently, investigators have revealed that the designed microfluidics-assisted H_2_S-releasing aspirin derivative (ACS14)-containing large porous microspheres showed promising potential as an inhaled and efficacious H_2_S donor in treating MCT-induced PH [58]. In addition, a variety of H_2_S donors have been developed. Naturally occurring donors include diallyl sulfide, diallyl disulfide, and diallyl trisulfide, while synthetic H_2_S donors consist of the following kinds: hydrolysis-triggered donors consisting of Lawesson’s reagent and derivatives, as well as dithiolthiones; thiol-triggered donors comprised of N-benzoylthiobenzamides, acyl perthiols, dithioperoxyanhydrides, polysulfides, arylthioamides, and S-aroylthiooximes; light-triggered donors which include geminal-dithiols, ketoprofenate photocages, and α-thioetherketones; enzyme-triggered donors; and finally, dual carbonyl sulfide/H_2_S donors consisting of N-thiocarboxyanhydrides and self-immolative thiocarbamates [56]. The clinical significance of H_2_S clinical translation and its donor discoveries in the treatment of PH merit interdisciplinary studies. 

## Data Availability

Not applicable.

## Abbreviations

ANP: atrial natriuretic peptide; CHD: congenital heart disease; CGRP: calcitonin gene-related peptide; CO: carbon monoxide; COPD: chronic obstructive pulmonary disease; COX-2:cyclooxygenase-2; CSE: cystathionine-γ-lyase; ECM: extracellular matrix; EndMT: Endothelial-to-mesenchymal transition; ET-1: endothelin-1; MCT: monocrotaline; H_2_S: hydrogen sulfide; Hcy: homocysteine; HO: heme oxygenase; HPH: hypoxic pulmonary hypertension; IKKβ: inhibitor of κB kinase subunit β; NF-κB: nuclear factor kappa B; NO: nitric oxide; NOX4: nicotinamide adenine dinucleotide phosphate oxidase 4; PAMP: pro-adrenomedullin peptide; PAP: pulmonary artery pressure; PASMC: pulmonary artery smooth muscle cell; PASP: pulmonary artery systolic pressure; PH: pulmonary hypertension; PGI2:prostaglandin; ROS: reactive oxygen species; SO_2_: sulfur dioxide; VSMC: vascular smooth muscle cell.
